# Interventional Retrospective Case Series of Patients Undergoing Treatment Intervals of More Than Twenty-Four (24) Weeks With Faricimab

**DOI:** 10.1155/crop/8843375

**Published:** 2025-02-14

**Authors:** Adrian Babel, Eric K. Chin, David Almeida

**Affiliations:** ^1^Wright State University Boonshoft School of Medicine, Dayton, Ohio, USA; ^2^Erie Retina Research, Department of Clinical Research, Erie, Pennsylvania, USA; ^3^Retina Consultants of Southern California, Redlands, California, USA; ^4^Loma Linda Eye Institute, Veterans Affair Hospital, Loma Linda, California, USA

**Keywords:** diabetic macular edema, faricimab, neovascular age-related macular degeneration

## Abstract

**Purpose:** The aim of this study is to evaluate the efficacy and safety of intravitreal faricimab dosing interval at and beyond 24 weeks in patients with diabetic macular edema (DME) and neovascular age-related macular degeneration (nAMD).

**Methods:** This study is a retrospective case series of eight patients with persistent DME and nAMD who received intravitreal faricimab at and beyond the 24-week (6-month) dosing interval regimen.

**Results:** The majority of patients experienced an improved mean best-corrected visual acuity (BCVA) of 9.9 letters; congruent anatomical improvement (mean central macular thickness (CMT)) decrease of 44 *μ*m on optical coherence tomography (OCT) is demonstrated at 6 months despite extended faricimab dosing intervals.

**Conclusions:** Extended intravitreal faricimab dosing intervals at and beyond 24 weeks maintained visual and anatomical outcomes in patients over 1 year. This suggests the feasibility of personalized extended dosing tailored to each patient's disease activity, potentially reducing treatment burden.

**Summary**

• Diabetic macular edema (DME) and neovascular age-related macular degeneration (nAMD) are two major ophthalmic diseases that impact central vision and typically require treatment with intravitreal injections.
• Short intravitreal treatment intervals place significant burden on patients and clinics.
• Are extended (24 weeks or more) treatment intervals with faricimab safe and effective for treating DME and nAMD?
• Faricimab treatment intervals at 24 weeks or more is safe and effective for maintaining or improving best-corrected visual acuity (BCVA) and central macular thickness (CMT) in patients with DME or nAMD.
• Durability of faricimab may allow clinicians to tailor treatment intervals at and beyond 24-week intervals for each patient's disease activity.
• This study presents findings that could transform the treatment paradigms for DMA and nAMD, potentially reducing treatment burden.

## 1. Introduction

The prevalence of DME, a principal cause of visual impairment among diabetic patients, has been a growing concern, affecting an estimated 21 million individuals globally as of 2008 [1, 2]. This condition, present in 2.7%–11% of those with diabetic retinopathy, is poised to escalate in tandem with the diabetes epidemic, which has already impacted approximately 537 million adults in 2021 [3, 4]. Projections suggest a surge to 783 million by 2045, potentially increasing the incidence of clinically significant DME to 28.6 million adults [3, 4]. The multifaceted nature of DME pathogenesis culminates in vision loss primarily due to the accumulation of submacular fluid near the fovea, driven by abnormal microvascular changes [3].

Concurrently, age-related macular degeneration (AMD), which affected over 3 million Americans in 2020, remains the leading cause of irreversible blindness in individuals over 60 [5, 6]. AMD manifests in two forms: nAMD and nonneovascular, with nAMD characterized by the emergence of new, aberrant blood vessels that penetrate the retina and threaten its structural integrity [7]. Without timely intervention, fluid leakage, hemorrhages, and subsequent retinal damage from these vessels can lead to significant vision impairment [7].

The advent of intravitreal anti-vascular endothelial growth factor (anti-VEGF) injections marked a significant breakthrough in the management of DME and nAMD, enhancing patient outcomes through effective and safe vision preservation [7, 8]. Nonetheless, the frequent administration required by anti-VEGF therapies poses a considerable burden on patients [7–9]. Recent investigations have identified angiopoietin-2 (Ang-2) as a critical factor in the pathophysiology of vasculopathy-related vision threats, often coexpressed with VEGF under pathological conditions and contributing to vascular instability and increased exudation [10].

Enter faricimab: As of January 2022, this novel bispecific monoclonal antibody has received FDA approval for concurrently inhibiting both VEGF and Ang-2 pathways [11]. Faricimab's dual-targeting approach offers a more comprehensive blockade against neovascularization while promoting vascular stability [12–15]. This was evidenced in the Phase III TENAYA and LUCERNE trials for nAMD and the YOSEMITE and RHINE trials for DME, which collectively demonstrated faricimab's noninferiority to aflibercept—a standard anti-VEGF therapy—in terms of treatment efficacy and safety, with the added advantage of extended dosing intervals [12–15].

These pivotal trials have shown that nearly 80% of patients receiving faricimab could maintain extended dosing intervals without compromising visual acuity gains or anatomical outcomes when compared to more frequent aflibercept injections [12–15]. The potential to extend treatment intervals beyond the current 16-week maximum presents an opportunity to alleviate the treatment burden for patients [7, 8].

This paper is aimed at delving into the clinical implications of these findings by examining case series that explore the feasibility and efficacy of extending faricimab dosing intervals even further, focusing on intervals at and beyond every 24 weeks. Such an extension could significantly reduce the frequency of treatments, thereby lessening patient burden and potentially reshaping the management paradigm for DME and nAMD.

## 2. Methods

### 2.1. Study Design and Participants

This retrospective case series included eight patients with DME and nAMD who received intravitreal faricimab injections with dosing intervals administered at and beyond 24 weeks. Eligibility criteria included a minimum age of 18 years, a diagnosis of DME or nAMD, and a history of at least two previous anti-VEGF injections before the initiation of faricimab treatment.

### 2.2. Data Collection

Medical records from March 2023 to March 2024 were reviewed for patient demographics, baseline visual acuity, ocular history, faricimab injection dates, dosing intervals, and adverse events. Optical coherence tomography (OCT) images and fluorescein angiography were also analyzed to assess anatomical outcomes. The primary outcome measure was the change in the BCVA letter score from the initiation of faricimab treatment to the last follow-up visit. Secondary outcomes included changes in CMT on OCT and the number of patients who achieved dosing intervals at and beyond 24 weeks without experiencing a loss of more than five letters of BCVA or significant anatomical deterioration.

### 2.3. Treatment Protocol

Faricimab was initially administered according to the FDA-approved dosing regimen. Based on individual patient responses, treating physicians considered extending the interval between injections.

Extension criteria to and beyond 24 weeks included the following:

- Absence of intraretinal or subretinal fluid on OCT for at least two consecutive visits
- Stable or improving BCVA with no loss more significant than five letters between visits
- No new hemorrhages on clinical examination
- No evidence of disease activity on fluorescein angiography if performed
- Minimum of three consecutive stable visits at the current interval before extending

Patients underwent monitoring every 4–8 weeks during the extension process. Any disease reactivation prompted immediate interval reduction.

### 2.4. Data Analysis

Descriptive statistics summarized patient demographics and baseline characteristics. Changes in BCVA and CMT before and after interval extension were compared using the Wilcoxon signed-rank test due to the small sample size and nonnormal distribution of differences between paired observations. A *p* value < 0.05 was considered statistically significant. All statistical analyses were performed using SPSS software (Version 26.0, IBM Corp).

### 2.5. Ethical Considerations

This study adhered to the tenets of the Declaration of Helsinki and was approved by the Institutional Review Board (IRB) at the hosting institution. Due to the retrospective nature of the study, the IRB waived informed consent. Patient confidentiality was maintained throughout the study by deidentifying all personal health information.

## 3. Results

In this retrospective case series, we identified eight patients with DME and nAMD who received intravitreal injections of faricimab at and beyond 24-week intervals, with some patients receiving injections as infrequently as every 40–44 weeks. The patients were closely monitored for changes in visual acuity and anatomical outcomes over a follow-up period of at least 1 year. Patient demographics include ages ranging from 56 to 85 years (mean = 72, SD = 10.94), four females (50%), four males (50%), three OS (37.5%), five OD (62.5%), three DME (37.5%), and five nAMD (62.5%). Three cases (37.5%) were at 24-week dosing intervals, one case (12.5%) was at 28-week intervals, one case (12.5%) was at 32-week intervals, two cases (25%) were at 40-week intervals, and one case (12.5%) was at 44-week interval. Six cases were excluded from OCT CMT analysis due to a lack of baseline data.

Seven out of eight (87.5%) patients maintained stable or improved visual acuity even with extended faricimab dosing intervals, with a mean improvement in BCVA letter score from baseline of 9.9 letters and mean LogMAR decrease of 0.197 (SD = 0.395) at 1 year despite lengthened injection intervals. Similarly, anatomical outcomes remained well-controlled in all patients, with a reduction of fluid on OCT with intervals at and beyond 24 weeks between faricimab doses. We excluded six cases from this analysis due to no baseline data. We observed a mean OCT CMT decrease of 44 *μ*m (SD = 43.035 *μ*m). One case worsened the BCVA letter score at 24 weeks but exhibited CMT improvement on OCT. No new safety signals arose during extended dosing intervals compared to standard q16 week faricimab treatment.

## 4. Discussion

This real-world case series provides promising early evidence supporting the feasibility of extending faricimab dosing intervals substantially longer than the currently approved 16-week interval in select patients with DME and nAMD. Our finding of maintained visual and anatomical outcomes even with treatment intervals extended to 40–44 weeks in some patients could represent a major advance towards reducing patient burden.

By showing that stable vision can be sustained in many patients on injection intervals far longer than prior anti-VEGF agents, this study suggests that faricimab's durability may enable a personalized treatment approach based on each patient's disease activity rather than a fixed schedule. If supported by further studies, this could transform treatment paradigms for retinal vascular diseases by lessening treatment frequency when disease stability permits while still allowing intensification as needed.

This study has several significant limitations. The small sample size (*n* = 8) limits statistical power and generalizability. Missing baseline OCT data in six cases impacts our ability to fully assess anatomical outcomes, though clinical outcomes remained favorable in these patients. As a retrospective analysis, selection bias may have influenced which patients received extended intervals. Additionally, our follow-up period may be insufficient to detect late disease recurrence.

In some cases, the absence of baseline OCT data represents a significant limitation in evaluating anatomical outcomes. However, the positive visual results in these patients support the potential viability of extended dosing intervals. The favorable BCVA outcomes, even without complete anatomical data, suggest clinical benefit, though we acknowledge the need for complete datasets in future studies to validate these findings fully.

More extensive prospective studies with complete imaging data and longer follow-up are needed to validate these preliminary findings before broadly implementing extended dosing intervals in clinical practice. Nonetheless, this case series paves the way for reassessing the feasibility of substantially extending treatment intervals in the faricimab era.

## 5. Conclusions

This retrospective case series provides early evidence that extending the dosing interval of intravitreal faricimab beyond the currently approved 16 weeks may be feasible and maintain visual and anatomical outcomes in select patients with DME and nAMD. The findings suggest that faricimab's durability could enable personalized extended dosing intervals guided by each patient's disease activity rather than a fixed schedule. This can transform treatment paradigms by reducing patient burden when disease stability allows while permitting treatment intensification as needed. Further research through controlled studies is warranted to confirm these findings in larger patient groups, better define suitable candidates, and determine the maximal duration of faricimab activity in quiescent DME and nAMD. Still, this study represents an essential step towards establishing the possibility of substantially reducing treatment frequency in the faricimab era to address a significant unmet need for these patients.

## Data Availability

The data that support the findings of this study are available from the corresponding author upon reasonable request.
